# The Problems That Interest Motherhood

**Published:** 1902-09

**Authors:** W. H. Felton

**Affiliations:** Cartersville, Ga.


ATLANTA

### Journal-Record of Medicine.

Successor to Atlanta Medical and Surgical Journal, Established 1855, and Southern Medical Record, Established 1870.

VOL. IV. SEPTEMBER, 1902. NO. 6.

BERNARD WOLFF, M.D., EDITOR, Nos. 319–20 Prudential.

Published Monthly.

M. B. HUTCHINS, M.D., BUSINESS MANAGER, No. 64 Marietta St.

### ORIGINAL COMMUNICATIONS.

### THE PROBLEMS THAT INTEREST MOTHERHOOD.*

BY MRS. W. H. FELTON,
CARTERSVILLE, GA.

My appearance before this audience to-day stands for no intimate acquaintance with the science of medicine, or knowledge of the questions which you discuss so ably. I am only a learner—and my eager interest in these matters no doubt influenced your President to extend to me an invitation to appear and explain my reasons for this interest. Three years ago I made an address on this subject before the Woman's Club of Atlanta, and in 1897 sent a paper to be read before the Mother's Congress in Washington City. My greatest regret is my inability to awake a lively interest in the minds of the mothers of the country on this and kindred subjects; because I feel sure if the subject could be pre-

*Read before the Georgia Sociological Society, Atlanta, June, 1902.

sented in force and pertinence there would be an awakening that would show itself in every home in this broad land of ours.

I agree with your President, that all human life is influenced by heredity; by training and environment in the order here named. Heredity comes first. Mothers should have some conception of the part they play in life's drama, when every child must have a mother, and when every child's life is unavoidably shaped by the mother's nature, her health, her tempers and idiosyncrasies. She herself is the repository of the ills and benefits resulting from close relation to her ancestry—those who handed them down to her. There are legacies handed down—bequeathed—that would shock and startle many of us if we could trace the end back to the beginning.

But our young women, about to step into matrimony, know less of themselves and what is involved in parental relations, than any other subject heretofore considered by them. Their previous education is all away from the subjects rather than towards them. Children are being born into this world every day, every hour, aye, every minute, and I feel sure that nine tenths of the mothers know less about the influences that will make or mar the physical, mental and moral destiny of those they bring into the world than any other subject that interests them. This ignorance is alarming. When stock-raisers, fruit-growers, seedsmen and everybody interested in such business matters are so scrupulously exact in securing proper reproduction, the destinies of their own children and grandchildren are put aside in the most indifferent way; and alliances are contracted in the most haphazard style. Truly, marriage is a lottery.

People move into new neighborhoods; settle in hard localities; invest their money and spend their time in getting money, when their children and grandchildren have scarcely a thought as to proper associates and those they will mate with, because they will mate at mating time; and the mates will be found generally where they chance to be associated. This is the rule—almost without exception.

If a piece of real estate is purchased, a lawyer is hired to trace the title, and the utmost care is taken to be sure, before the money is paid over; but when the daughter falls in love with an unknown lover, and maybe marries a whited sepulcher, with a fair outside, but full of inherited rottenness and dead men's evils, what comfort or happiness or peace of mind will attend the money, after such a disaster?

Why is it that the world does not recognize the fact, that motherhood and all the problems of human life are indissolubly connected and cannot be divorced, since every human being must have its mother?

With the snows of sixty winters drifting down on my head, and my steps going down the sunset slope of life, I come to you, to add my word of approval to your undertaking, and to beg you to so broaden the scope of your organization that intelligent womanhood may be instructed, entreated and warned on the subjects in which their interest is so great, with influences so potential.

For two decades I have lifted my small voice in Georgia towns and cities to plead against the crime of forcing little children, without their own consent, into drunken homes and entailing on their little lives the curse that travels along with inebriety and debauchery. I have been called "a fanatic, a crank, strong-minded, out of my sphere," and many names of that sort, because I cried aloud for home protection to the mothers of such children; but standing as I do in the sight of God and in the presence of this distinguished company, I affirm, as my honest conviction, that no greater service can be done for the human family—without regard to sex—than the protection of unborn children from the evils—inherited evils—that go with drunkenness; and this protection must come through the united efforts of the mothers and fathers of these children.

How many families can you call to mind, who, after twenty-five years of marital union, can present a clean bill of health—moral, mental and physical? Without deformity, insanity, drunkenness, immorality and crime? Tons of writing-paper have been devoted to the proper training of children; and pulpits have thundered for a full century on the curses that follow to the third and fourth generation; but how little has been said or written or preached about the proper training of fathers and the needful instruction of mothers in the greatest calling ever followed or pursued under the shining dome of Heaven!

Mothers hold the race-endowing function in themselves. The Lord Almighty decreed it. This function is at once the greatest mystery and the grandest work ever confided to human kind; but nine tenths of the women—may I not say ninety hundredths—know no more of the dangers that attend the destinies of the unborn child, or of their own intimate relation to reproduction of their own kind, than the birds in the air or fishes in the sea. I blame mothers for much of this ignorance—but much of it also belongs to the apathy and indifference of the public. No stream rises higher than its source. The mother can only give her child what she herself possesses. Oh! that we might reckon in the list of virtues the uncommon attribute of common sense on these subjects.

Mothers have been trained, unfortunately, to a system of false modesty and unwholesome avoidance of this and kindred subjects. Why should not they instruct their daughters, aye, their sons, as the king's chamberlain tutored Esther before she was wedded to Ahasuerus? Oh! it is pitiful to know that a young girl is pushed out of the home nest to flutter in dismay and too often to perish on the ground at last! How is she to know without a teacher, and what teacher so proper, so tender, so sympathetic and so loyal as her own parents?

There were thirteen thousand divorce cases in New York city alone in the year 1897. That tells a tale of failure, defeat, disappointment, mismating, that needs no comment; and it is so all over the land.

When the census of 1890 was taken there were 700,000 defectives in the United States—blind, deaf, deformed, insane, imbecile, epileptic, etc. That is another story that needs no explanation in this presence.

I believe as firmly as my mind can grasp the condition, that the negro's education in books has been largely unproductive of good results, because it antedated the proper training of the mothers in their lewd homes.

This land is burdened with convicts and ex-convicts, the latter without character or credit, after they are turned loose on the community. These lewd homes are continual crime-promoters. They pull down faster than book education can build up. If I could only whisper a word in Mr. Carnegie's willing ear, I'd say, spend some of your money on reformatories for ignorant women and girls, who should never be permitted to marry or propagate their kind until good character shall have been established.

This brings me to the question of indiscriminate marriage licenses. A health certificate should have been required a hundred years ago. No honorable man would be willing to enter the parental state when his own physical conditions failed to warrant reproduction under their influence and sway. Those who would disregard the restriction should be legally restrained, for public safety, in view of public expense.

Now that the State has assumed the burden of educating the child independent of its parents, it should be given discretionary powers to forbid the spawning of an interminable horde of defectives with inherited evils of mind and body. Begin at the beginning. Reform where it needs reformation. The running stream can be diverted when it is a brook. After it becomes the river, it must go its own way. Hereditary characteristics are transmitted. There is no question about it. Some weeks ago statistics were given in New York State as to the progeny of a woman who died in 1828. She had 800 known descendants. Nine tenths were criminals, as she had been a criminal; some hundreds were diseased in various ways; many were hung—some died serving life-sentences; and the State spent thousands upon thousands of dollars in convicting and punishing that woman's descendants. You all read her history, I presume. If her crime had made her immune to child-bearing, then she would have been less inimical to public safety and spared some physical suffering also. She was an unfit person to fling 800 of her own dangerous class upon the public purse, and her descendants were a curse to the population upon which she and those like her were permitted to spawn them. There can be no question as to the duty of the State to protect itself from this indiscriminate spawning, and to protect itself from indiscriminate marriage licenses. Your organization has authority to make itself heard before legislative committees. Why not inaugurate the movement yourselves?

I believe the State should require due notice to be given of an intended marriage. You all understand that you cannot buy a piece of land from an administrator of an estate without publishing it for weeks in a public gazette. He must get leave to sell and then give equal notice of proposed sale; but any chap that can beg or borrow a dollar and a half and will swear that the parties are of legal age, can go to the ordinary's office between dark and daylight, and buy a permit to marry a woman who may have 800 descendants, good, bad or indifferent, within her allotted period of reproduction. Fifty years from now public opinion will stand aghast at the liberty that is now allowed to lust and liquor. When a money purchase is involved (as the sale of land goes to show) you strain at gnats, gentlemen, but when the fate of unborn millions hangs in the balance you swallow camels, and never make a wry face at the swallow.

Motherhood is now asking itself why these things have been so long overlooked—why the protection to its high and holy calling has been so long delayed.

Public thought must be awakened! Agitation is imperative for these reforms. Nothing can be done until the people begin to think. Perhaps your are living ahead of your time. I feel sure I have been working with a small pick and shovel for twenty years at least, with small evidence of progress; but you know and I know that we are confronted with race problems and all other sorts of problems, all of which alarm the patriot and the tax-payer.

We are living in an era of mob violence which is ominous. We seem to hear the earth tremblings that may culminate in a San Domingo or a Mont Pierre disaster. May God help the helpless and innocent!

As I believe in eternal justice, truth and mercy, I do believe we must begin reforms at the starting place, and remodel our permits to marry, and permits to sell crime-promoters and stand firm like Zerubbabel in the rebuilding of our earthly Jerusalem, or we will, ere we know it, find a Mont Pelee right in our midst, with all that attends such an eruption in disaster.

To you, gentlemen, who are so closely in touch with the great problems that touch mother-life and mother-love, I bring this reminder to-day. Throw around this high and holy calling the protection it needs and would call for if it was awake to its danger. It is the hope of the nation. It is the anchor against mob violence. It is the home-life of America to-day that holds the ship to the safe points of the compass.

It is at the very foundation of race troubles. When colored homes are reformed from their profligate population, and clean living prevails rather than dissolute and shameless reproduction, we will see that it can only be done by shutting off opportunities for infanticide and indecent prostitution among the mothers of the colored race. But all the crime does not come from the submerged world. Like the frogs and flies in Egypt, sin, evil and moral disease go up into the palaces as well as the hovels. There must be a general provision to reach crimes against motherhood—no matter where discovered.

In the year 1874 the State had many less than four hundred convicts to turn over to the lease system. We established public schools a little before that time. We have run along the succeeding years with both systems in full blast—both costing a mountain of money. In 1901 we had four thousand negro convicts and misdemeanors, and about four hundred whites. Now what is the matter with us anyhow! Where will we end up at this gait we are going? If we had clear vision to the horizon we would see that one system tears down faster than the other can build up. Something is radically wrong somewhere.

I may be mistaken—I know I am liable to mistakes; but I do believe we should shut the door on the reproduction of the species that continues to fill chain-gangs, and apply some of this school money to reformatories where they do not marry and are not given in marriage until they can present a clean bill of moral health, at least.

An illegitimate child should send both parents to the rock-pile, and a seducer of innocence should have a life-term at hard work; fully as much penalty as the getting of money on false pretenses, by theft or by murder, because two lives have been sacrificed to the baser passions of the betrayer.

Oh ! that the world would wake up to this needed protection to the unborn child which is forced into life, without any consent of its own, and its very life made the football of the vicious and depraved progenitor !

I thank you very much for this courtesy and attention. May my words find an echo in every true man's and woman's heart that hears me to-day.

